# Necessity of concurrent chemotherapy in N2‐3 nasopharyngeal carcinoma treated with neoadjuvant chemotherapy of ≥3 cycles followed by intensity‐modulated radiotherapy

**DOI:** 10.1002/cam4.2179

**Published:** 2019-04-21

**Authors:** Hui Chang, Liang Peng, Ya‐Lan Tao, Chen Chen, Wei‐Wei Xiao, Yong‐Hong Hu, Yuan‐Hong Gao

**Affiliations:** ^1^ Department of Radiation Oncology Sun Yat‐Sen University Cancer Center, State Key Laboratory of Oncology in South China, Collaborative Innovation Center for Cancer Medicine Guangzhou, Guangdong China

**Keywords:** concurrent chemotherapy, N2‐3 disease, nasopharyngeal carcinoma, survival, toxicities

## Abstract

Concurrent chemotherapy (CCT) is used in locally advanced nasopharyngeal carcinoma (NPC) for improved local control, which could also be achieved by intensity‐modulated radiotherapy (IMRT). And for N2‐3 NPC, distant metastasis is the more important cause of death. This study aims to evaluate the value of CCT in N2‐3 NPC when neoadjuvant chemotherapy (NACT) of sufficient cycles is performed to eradicate distant metastasis. It enrolled 959 patients diagnosed with TxN2‐3M0 NPC from July 2011 to December 2015 and treated with NACT of 3‐4 cycles and IMRT. A propensity score matching (PSM) was made between patients treated with and without CCT (called the CCT and non‐CCT groups, respectively), using a series of clinical characteristics (age, gender, T stage, N stage, NACT regimen, and EBV DNA) as covariates. After PSM, the two groups of patients were compared on survivals and acute toxicities. The results indicated that no difference was seen in the overall, disease‐free, recurrence‐free or metastasis‐free survivals between the two groups. But compared with the CCT group, the non‐CCT group had a lower patient proportion of myelosuppression, nausea/vomiting, oral mucositis, cervical dermatitis, xerostomia, and grade 3/4 myelosuppression and oral mucositis (all *P* values were <0.001). Hence, CCT appeared to bring more acute toxicities, instead of survival benefit, to N2‐3 NPC patients treated with NACT of ≥3 cycles and IMRT. It should be used with cautions in these patients.

## 1 INTRODUCTION

Nasopharyngeal carcinoma (NPC) is a common malignancy in South China.^1^ Radiotherapy is now the primary management for NPC of all stages. For patients with locally advanced (stage III‐IVB) disease, concurrent chemotherapy (CCT) is recommended to enhance radiosensitivity and local control.^2^ Since distant metastasis (DM) gradually becomes the major cause of treatment failure,^3^ neoadjuvant chemotherapy (NACT) followed by CCT has recently been tried as a new chemotherapy sequence in these patients. However, the results appeared to be inconsistent.^4^, ^5^, ^6^, ^7^, ^8^

Approaches have also been made to screen out patients really at high risk of DM and in need of NACT. N stage emerged as one of the most important factors for predicting DM, in a series of prognostic studies.^9^, ^10^, ^11^ Namely, NPC of late N (N2‐3) stages might be an indication for NACT. Furthermore, studies by Peng et al and us found that NACT of ≥3 cycles might be more effective in eradicating DM and improving overall survival (OS) for N2‐3 NPC.^12^, ^13^

In spite of improved local control, CCT could simultaneously increase incidence of severe acute and late toxicities.^14^, ^15^ Actually, intensity‐modulated radiotherapy (IMRT) was reported to bring a satisfactory local control, even without CCT in some studies.^15^, ^16^ Hence, we hypothesized that CCT might not be necessary for N2‐3 NPC in the era of IMRT, especially under NACT of sufficient cycles.

Here we conducted a prospective observational study to evaluate the impact of CCT on local recurrence (LR), DM, OS and acute toxicities, in a cohort consisting of 959 patients diagnosed with N2‐3 NPC and treated with NACT of ≥3 cycles followed by IMRT.

## 2 MATERIALS AND METHODS

### 2.1 Patient selection

Patients with pathologically diagnosed NPC from July 1, 2011 to December 31, 2015 in our hospital were initially considered. A patient would be enrolled and prospectively observed if he or she had: (a) age ≤70 years old; (b) TxN2‐3M0 diseases; (c) NACT of ≥3 cycles; (d) irradiation with IMRT. The exclusion criteria included: (a) Karnofsky performance score ≤70; (b) severe heart, lung, liver or kidney dysfunctions unsuitable for radiotherapy; (c) previously treated NPC; (d) prior history of other malignancies, chemotherapy or radiotherapy; (e) DM before or during radiotherapy; (f) application of adjuvant chemotherapy or monoclonal antibody. This study was approved by the Institutional Review Board of our hospital. Written informed consents were obtained from all individual participants before treatment.

### 2.2 Diagnostic work‐up

Before treatment, each patient received electronic nasopharyngoscope, magnetic resonance imaging (MRI) of head and neck, thoraco‐abdominal computed tomography (CT), whole‐body bone scan (or positron emission tomography). Serum level of Epstein‐barr virus (EBV) DNA was performed before and after NACT. A patient's stage was determined based on the Union for International Cancer Control/American Joint Cancer Committee TNM classification. For convenience of analysis, all the patients were restaged according to the 8th edition.

### 2.3 Treatment strategy

All the enrolled patients were treated with NACT of 3‐4 cycles, followed by IMRT‐based radiotherapy plus CCT or not. NACT was performed once per 3 weeks, with one of the following regimens: (a) docetaxel + cisplatin + fluorouracil (TPF); (b) cisplatin + fluorouracil (PF). CCT was administered with single‐agent cisplatin regimen, weekly or every 3 weeks throughout the whole procedure of radiotherapy. According to clinical practice guidelines of our hospital, CCT would be omitted if a patient had one of the three situations: (a) size of all his or her positive lymph nodes reduced to <1 cm after NACT; (b) grade 3/4 myelosuppression (MS) happened twice during NACT; (c) he or she refused to receive CCT. The radiotherapy technique was IMRT, whose target delineation and dose prescription were based on the International Commission on Radiation Units and Measurements Report 83. The treatment‐related acute toxicities were graded according to the Common Terminology Criteria for Adverse Events version 4.0.

### 2.4 Follow‐up

Patients were followed up after treatment through outpatient interview, every 3‐6 months in the first 3 years. The main contents of outpatient interview included complete physical examination, thoraco‐abdominal CT, head and neck MRI, serum EBV DNA assessment, and annual whole‐body bone scan (or positron emission tomography). Follow‐up was performed through outpatient interview or telephone every 6‐12 months in the 4th and the 5th years, and every 12 months thereafter until death from NPC or June 30, 2018, whichever came first. Causes of deaths were confirmed by death certificates.

The primary endpoint of this study was OS, which was defined as the proportion of the patients who survived after a defined time period from pathologic diagnosis. The secondary endpoints included recurrence‐free survival (RFS), metastasis‐free survival (MFS), and disease‐free survival (DFS). These three endpoints were defined as the proportion of the patients who had no corresponding events after a certain time period from diagnosis. The events for RFS and MFS were local recurrence (LR) and DM, respectively. And the events for DFS included death, LR and DM. And we also used patient proportion of the common toxicties as secondary endpoints, including MS, nausea/vomiting (NV), oral mucositis (OM), cervical dermatitis (CD), xerostomia (XS).

### 2.5 Statistical analysis

Propensity score matching (PSM) were performed to balance baseline clinical characteristics between the patients treated with and without CCT (called the CCT and the non‐CCT groups, respectively). It was based on a logistic regression and used a matching ratio of 1:3. The covariates included age, gender (male vs female), T stage (T3‐4 vs T1‐2), N stage (N3 vs N2), NACT regimen (PF vs TPF), and EBV DNA (detectable vs undetectable) between NACT and radiotherapy. The median value of age was used as its cutoff value. Patients’ baseline characteristics were compared through a Chi‐square test, both before and after PSM to confirm the balancing effects of PSM.

A Kaplan‐Meier approach was performed to calculate the survivals of the post‐PSM cohort. The cases without death, LR or DM until June 30, 2018, and those lost to follow‐up were regarded censored. Survival difference between the non‐CCT and the CCT groups was assessed by a log‐rank test. And CCT was also analyzed for its correlation with the covariates in PSM procedure, by using the Spearman's method. Finally, toxicities in these two groups were compared, also through a Chi‐square test.

The whole procedure of statistical analysis was done by IBM SPSS Statistics 23.0 (IBM Co., Armonk, NY**)**. A difference with a two‐sided *P* value <0.05 was considered to be statistically significant.

## 3 RESULTS

### 3.1 Patient enrollment

**Figure 1:**
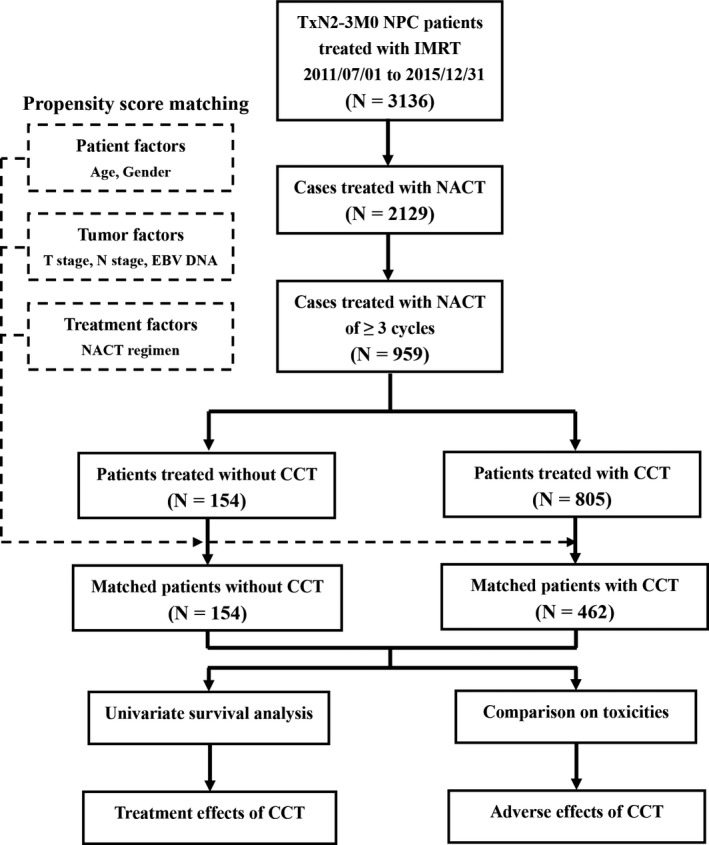
Procedure of enrollment and analysis. NPC, nasopharyngeal carcinoma; IMRT, intensity‐modulated radiotherapy; NACT, neoadjuvant chemotherapy; CCT, concurrent chemotherapy; EBV, Epstein‐barr virus

Between July 2011 and December 2015, a total of 3136 patients were diagnosed with N2‐3 NPC and irradiated with IMRT in our hospital. Of those, 2129 patients were managed with NACT before radiotherapy. And among these patients, 959 cases received NACT of ≥3 cycles and eligible for analysis, including 219 patients receiving NACT of 4 cycles. No patient received NACT of >4 cycles. According to record of CCT, 154 and 805 cases were divided into the non‐CCT and the CCT groups, respectively. After PSM, the case numbers of these two groups were 154 and 462, respectively. The procedure of this study was summarized as Figure 1.

**Figure 2:**
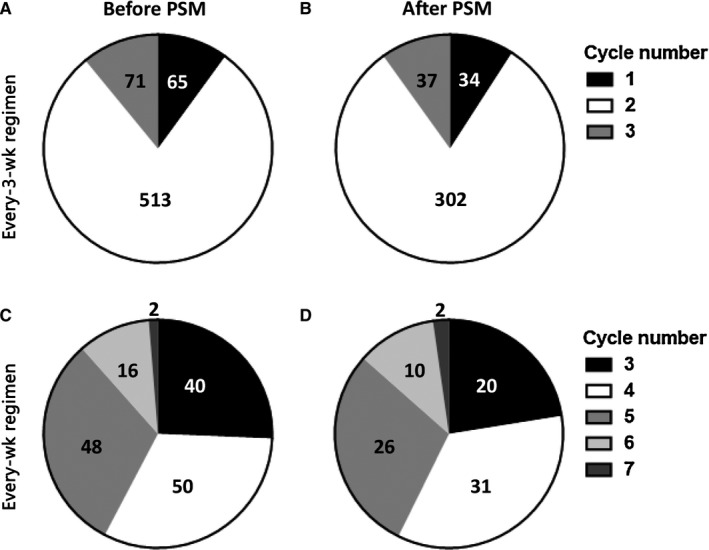
Numbers of patients receiving different cycles of cisplatin chemotherapy. A, Before propensity score matching (PSM), every‐3‐week regimen. B, After PSM, every‐3‐week regimen. C, Before PSM, every‐week regimen. D, After PSM, every‐week regimen

In the CCT group, a total of 649 and 156 patients were treated with cisplatin of every‐3‐week and every‐week regimens, before PSM. After PSM, the case numbers were 373 and 89, respectively. The actual cycle numbers that the patients received were summarized in Figure 2.

### 3.2 Clinical profile

The baseline clinical profiles before and after PSM were shown in Table 1. Before PSM, the age of the patients ranged from 10 to 73 (median, 44) years old. Thus, the cutoff value of age was 44 (≥45 vs ≤44) years old. Compared with the CCT group, the non‐CCT group had more cases with age ≥45 years old (56.5% vs 43.6%, *P* = 0.003), less cases with detectable EBV DNA after NACT (70.8% vs 84.2%, *P* < 0.001) and more cases receiving PF regimen (53.2% vs 30.1%, *P* < 0.001). Yet, PSM balanced all the baseline characteristics between these two groups.

**Table 1:** Baseline clinical profiles of the patients

Characteristics	N2‐3 NPC patients treated with IMRT and NACT of ≥3 cycles					
	Before PSM			After PSM		
	CCT		*P* value	CCT		*P* value
	No (N = 154)	Yes (N = 805)		No (N = 154)	Yes (N = 462)	
Age/y old						
≥45	87 (56.5%)	351 (43.6%)	0.003**	87 (56.5%)	246 (53.2%)	0.484
≤44	67 (43.5%)	454 (56.4%)		67 (43.5%)	216 (46.8%)	
Gender						
Male	115 (74.7%)	640 (79.5%)	0.180	115 (74.7%)	343 (74.2%)	0.915
Female	39 (25.3%)	165 (20.5%)		39 (25.3%)	119 (25.8%)	
T stage						
T3‐4	108 (70.1%)	617 (76.6%)	0.085	108 (70.1%)	359 (77.7%)	0.057
T1‐2	46 (29.9%)	188 (23.4%)		46 (29.9%)	103 (22.3%)	
N stage						
N3	57 (37.0%)	359 (44.6%)	0.082	57 (37.0%)	170 (36.8%)	0.962
N2	97 (63.0%)	446 (55.4%)		97 (63.0%)	292 (63.2%)	
EBV DNA						
Detectable	109 (70.8%)	678 (84.2%)	<0.001**	109 (70.8%)	357 (77.3%)	0.104
Undetectable	45 (29.2%)	127 (15.8%)		45 (29.2%)	105 (22.7%)	
NACT regimen						
PF	82 (53.2%)	242 (30.1%)	<0.001**	82 (53.2%)	208 (45.0%)	0.077
TPF	72 (46.8%)	563 (69.9%)		72 (46.8%)	254 (55.0%)	

Abbreviations: CCT, concurrent chemotherapy; EBV, Epstein‐barr virus; IMRT, intensity‐modulated radiotherapy; NACT, neoadjuvant chemotherapy; NPC, nasopharyngeal carcinoma; PF, cisplatin + fluorouracil; PSM, propensity score matching; TPF, docetaxel + cisplatin + fluorouracil.

* *P* < 0.05, **P < 0.01.

### 3.3 Patients’ survival

After a median follow‐up time of 41.8 (range, 6.2‐75.7) months, 12 out of the 616 patients (1.9%) in the post‐PSM cohort were lost to follow up. Until June 2018, there were totally 107 deaths (17.3%), 64 LRs (10.4%) and 123 DMs (20.0%). Among the 100 cancer‐related deaths, 91 cases (91.0%) harbored DM, and 34 cases (34.0%) harbored LR.

**Figure 3:**
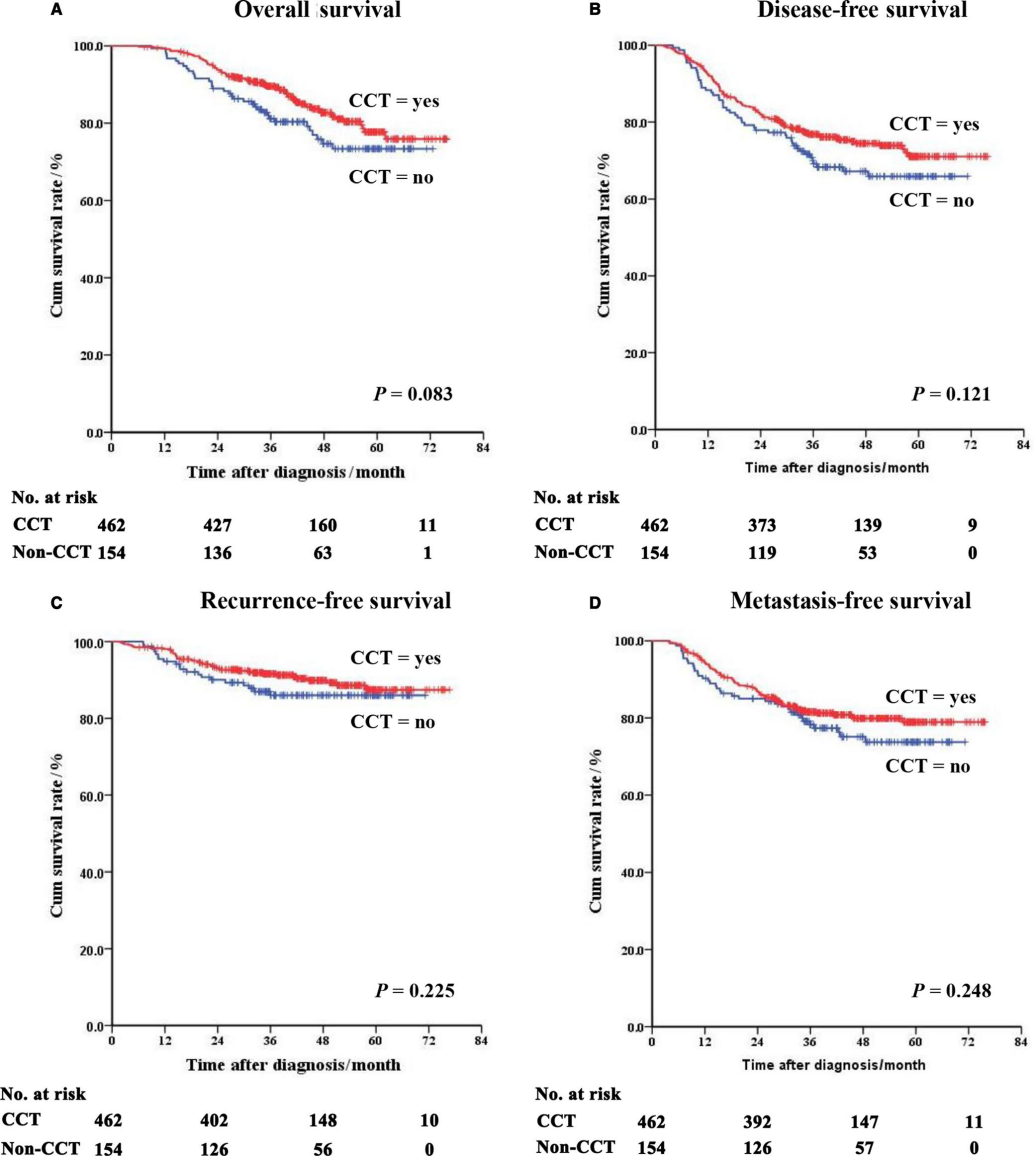
Survival curves of the matched patients. A, Overall survival; B, Disease‐free survival; C, Recurrence‐free survival; D, Metastasis‐free survival. CCT, concurrent chemotherapy

The survival curves of the non‐CCT and the CCT groups were summarized as Figure 3. The 5‐year estimated OS, DFS, RFS and MFS of the non‐CCT group were 73.4%, 65.9%, 86.0% and 73.8%, respectively. And the figures of the CCT‐group were 77.7%, 71.1%, 87.4% and 78.9%, respectively. There was no survival difference between these two groups. The *P* values were 0.083, 0.121, 0.225 and 0.248, respectively.

**Figure 4:**
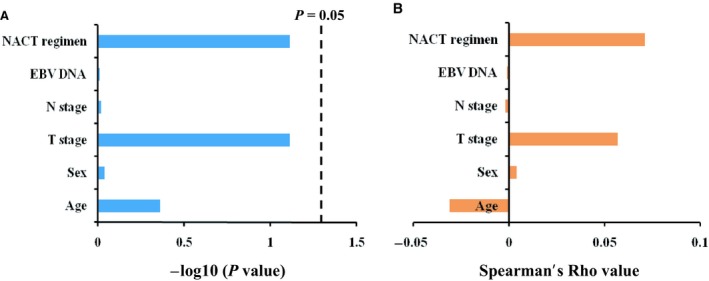
Correlation between concurrent chemotherapy and other clinical factors. A, Statistical significance for each factor; B, Spearman's Rho coefficient for each factor. NACT, neoadjuvant chemotherapy; EBV, Epstein‐barr virus

In addition, correlation analyses (Figure 4) showed that CCT had no correlation with other clinical factors, including age, sex, T stage, N stage, NACT regimen and EBV DNA (*P* values were 0.435, 0.915, 0.077, 0.962, 0.979 and 0.077, respectively). In other words, administration of CCT was not influenced by any one of these factors.

### 3.4 Acute toxicities

In the post‐PSM cohort, there were totally 195 (31.7%), 103 (16.7%), 253 (41.1%), 249 (40.4%) and 210 (34.1%) cases suffering from MS, NV, OM, CD and XS, respectively. The case numbers of grade 3/4 MS, NV, OM, CD and XS were 64 (10.4%), 5 (0.8%), 81 (13.1%), 11 (1.8%) and 2 (0.3%), respectively.

**Figure 5:**
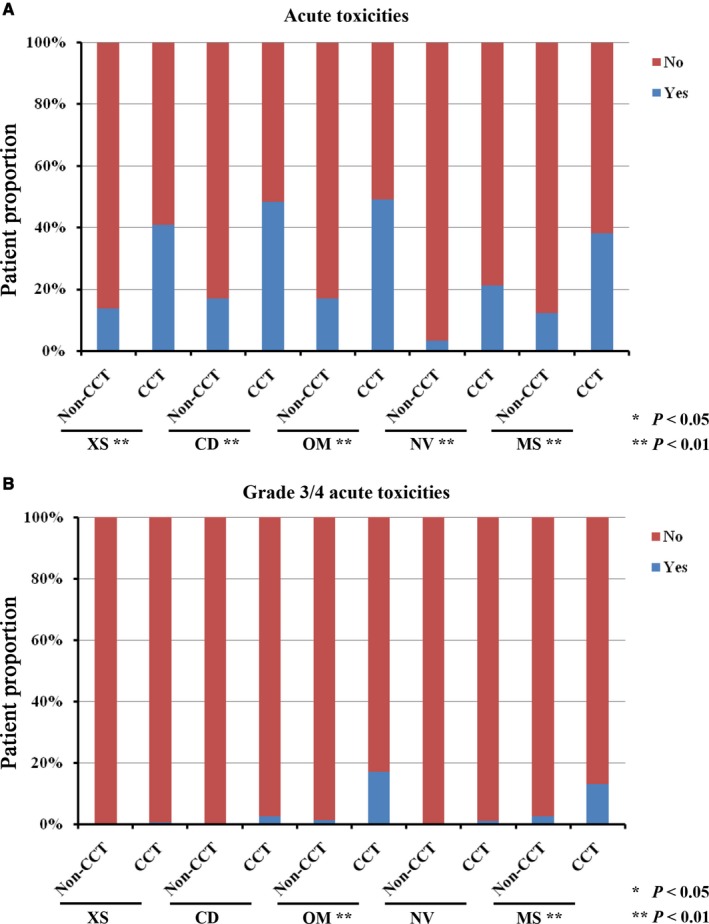
Patient proportions of acute toxicities in the matched patients. A, Toxicities; (B) grade 3/4 toxicities. CCT, concurrent chemotherapy. MS, myelosuppression; NV, nausea/vomiting; OM, oral mucositis; CD, cervical dermatitis; XS, xerostomia. *P < 0.05, **P < 0.01

Comparison on acute toxicities referred to Figure 5. Compared with the CCT group, there were obviously less cases exhibiting MS (12.3% vs 38.1%), NV (3.2% vs 21.2%), OM (16.9% vs 49.2%), CD (16.9% vs 48.3%) and XS (13.6% vs 40.9%) in the non‐CCT group (all *P* values were < 0.001). When comparing grade 3/4 toxicities, the non‐CCT group also had lower patient proportions of grade 3/4 MS (2.6% vs 13.0%, *P* < 0.001) and OM (1.3% vs 17.1%, *P* < 0.001) than the CCT group. Difference of other grade 3/4 toxicities was not observed between these two groups.

## 4 DISCUSSION

In literature, our study is the first study focusing on therapeutic and adverse effects of CCT in N2‐3 NPC patients who received NACT of ≥3 cycles. In this study, we found that CCT did not provide benefit of 5‐year estimated OS, DFS, RFS or MFS to these patients. Instead, CCT had a potential risk to increase incidence of acute toxicities, particularly severe MS and OM. The results were based on a large cohort of patients treated uniformly with IMRT, the modern stream of RT technique. And PSM, an effective method in controlling selection bias,^17^ was applied to balance baseline characteristics of the CCT and the non‐CCT groups before comparison, including the known prognosticators of NPC. These strengths made our results reliable.

After a median follow‐up time of over 3 years, we showed a DM and LR rate of 20.0% and 10.4%, respectively. Moreover, 91.0% of the NPC‐related deaths had DM. By contrast, only 34.0% of them underwent LR It was in accordance with previous studies that DM, rather than LR, is the primary obstacle to treatment success in N2‐3 NPC patients.^9^, ^10^, ^11^, ^12^, ^13^ As we know, DM is mainly eradicated by chemotherapy. Therefore, it could naturally be speculated that prognosis of N2‐3 NPC is associated with dose intensity of chemotherapy. Actually, intensified chemotherapy has been reported in many studies to decrease DM and improve survival of locally advanced NPC. Wei et al first found in 214 patients with stage II‐IVA NPC that a cumulative cisplatin of >200 mg/m^2^ was related to an improved DFS and MFS.^18^ In a subsequent study by Guo et al, a cumulative cisplatin of >100 mg/m^2^ emerged to improve OS and MFS of NPC patients with stage II‐IVB disease.^19^ Favorable impact of sufficient cisplatin dose (>240 mg/m^2^) on patients’ DFS was also seen in a study by Peng et al^20^ However, some studies indicated that cisplatin dose >240 mg/m^2^ failed to further improve patient survival. These studies established the optimal dose interval as 160‐240 mg/m^2^.^21^, ^22^ In our hospital, the cisplatin dose of NACT was 60 mg/m^2^ and 80 mg/m^2^ in the TPF and the PF regimens, respectively.^4^, ^13^ So the cumulative cisplatin dose for the patients of our study could reach ≥180‐240 mg/m^2^, which was showed to be effective in controlling DM. Additional dose of cisplatin might be unnecessary, as what was seen in the survival analysis. There was merely a MFS difference of 5.1% (*P* = 0.248) and an OS difference of 4.3% (*P* = 0.083), between the CCT and the non‐CCT groups.

It was also noteworthy that the RFS difference between these two groups was only 1.4% (*P* = 0.225). As we mentioned above, IMRT could lead to ideal local control. Large‐scale retrospective studies showed that advent of IMRT made the 10‐year LR rate of NPC decrease to only 3.5%‐5.0%.^23^, ^24^ It is superior to conventional 2‐dimensional radiotherapy in precise dose delivery, which simultaneously realizes maximal tumor killing and protection of adjacent normal tissue. On the other hand, combination of radiotherapy and CCT resulted in more severe toxicities, even in the era of IMRT. Some toxicities, such as OM and bilateral hearing loss, strongly affect patients’ compliance during radiotherapy and their long‐term life quality.^14^, ^15^, ^25^ Because NACT seemed to be well tolerated by NPC patients,^6^, ^26^ some oncological physicians tried to omit CCT, especially when NACT was administered. Zhang et al reviewed 440 patients with stage II and T3M0N0 NPC and discovered that IMRT alone was not inferior to IMRT plus CCT in either local or distant control. But addition of CT brought 5% more grade 3/4 MS and NV, and 10% more OM.^14^ Nevertheless, omission of CCT was uncertain in NPC of more advanced (III‐IVB) stages. In a study by Sun et al, CCT was not a predictor for local control, MFS, DFS and OS in these patients. Similarly, it made incidence of all grade 3/4 toxicities rise from 21.5% to 44.9%.^27^ Oppositely, a meta‐analysis involving 15 studies, 1142 patients indicated that combination of CCT and IMRT was still responsible for a higher complete response rate and a longer OS, as well as a higher risk of grade 3/4 MS and OM.^28^ Comparison between NACT and CCT was first made by Yao et al in 214 patients with T3‐4N0‐1M0 NPC. Though equal therapeutic effects were observed, NACT plus IMRT decreased 10%‐15% of grade 3/4 adverse reactions, including MS, OM, XS and hearing loss.^15^ A study by OuYang et al achieved analogous results in NPC patients with stage II‐IVB disease.^29^ Lin et al and Liu et al further analyzed patients diagnosed with stage II‐IVB NPC and treated with NACT. The results of the 2 studies both indicated that after NACT, CCT significantly elevated incidence of severe toxicities (29.8%‐50%), rather than improving tumor control. Particularly grade 3/4 MS and OM, the incidence raised to 5.7 and 19.0 folds, respectively.^30^, ^31^ And considering the treatment and adverse effects of CCT in our study, it might be rational to omit CCT in N2‐3 NPC treated with sufficient intensity of NACT.

Indeed, there were three main limitations of this study. First, it was a single‐institutional study. Second, it was an observational study without random allocation of patients into the CCT and the non‐CCT groups. Third, it did not report late toxicities because it had mainly been designed to assess impact of CCT omission on treatment effects and tolerance. So we recommended the results be generalized after external validation or randomized controlled trials. And more detailed safety analysis might also be needed.

In conclusion, CCT failed to bring survival benefit in N2‐3 NPC treated with NACT of ≥3 cycles followed by IMRT. On the contrary, it was responsible for more severe acute toxicities. Therefore, it should be used with cautions in these patients.

## CONFLICT OF INTEREST

None declared.
